# Burden of measles in Nigeria: a five-year review of casebased surveillance data, 2012-2016

**DOI:** 10.11604/pamj.supp.2019.32.1.13564

**Published:** 2019-01-22

**Authors:** Baffa Sule Ibrahim, Rabi Usman, Yahaya Mohammed, Zainab Datti, Oyeladun Okunromade, Aisha Ahmed Abubakar, Patrick Mboya Nguku

**Affiliations:** 1 Center for International Health, Education and Biosecurity, University of Maryland Baltimore (CIHEB-UMB), Maryland; 2 Nigeria Field Epidemiology and Laboratory Training Program, Abuja, Nigeria; 3 Department of Medical Microbiology, Usmanu Danfodiyo University, Sokoto, Nigeria; 4 Bayero University, Kano, Nigeria; 5 Ahmadu Bello University, Zaria, Nigeria

**Keywords:** Disease outbreak, measles, Nigeria, vaccination

## Abstract

**Introduction:**

measles is a vaccine preventable, highly transmissible viral infection that affects mostly children under five years. We reviewed surveillance data on measles from Nigeria over a five-year period to highlights its burden and make recommendations for improvements.

**Methods:**

we conducted a secondary data analysis of measles specific Integrated Disease Surveillance and Response (IDSR) records of all states in Nigeria over a five-year period.

**Results:**

a total of 131,732 cases were recorded between January 2012 and September 2016. Most cases 57,892 (43.95%) were recorded in 2013 while the least 11,061 (8.4%) were recorded in 2012. A total of 817 deaths were recorded, with a case fatality rate (CFR) of 0.62%. The highest CFR (1.43%) was recorded in 2012 while the least CFR (0.44%) was recorded in 2016. Only 8,916 (6.7%) cases were confirmed by laboratory tests. The trend of measles cases followed the same pattern throughout the years under review, with cases peaking at March, then gradually reducing to lowest level at June, which was maintained throughout the rest of the year. States in northern region of Nigeria recorded the highest attack rate (Yobe: 480.29 cases per 100,000 population, Sokoto: 284.63 cases per 100,000 population and Katsina: 246.07 cases per 100,000 population) compared to States in the southern region (Rivers: 11.72 cases per 100,000 population and Akwa Ibom: 13.59 cases per 100,000 population). Conversely, States in the southern region recorded the highest CFR (Ebonyi: 13.43% and Rivers: 3.27%).

**Conclusion:**

measles infection remains a burden especially in the northern region of Nigeria. Although measles fatalities declined over the years, laboratory confirmation was sub-optimal. We recommended improvement on routine immunization and strengthening of regional laboratories diagnostic capacities, for successful eradication of measles from Nigeria.

## Introduction

Measles is a highly contagious viral disease caused by Morbillivirus; a member of the Paramyxovirus family, which is transmitted to a susceptible individual through aerosol or by direct contact [1]. The virus infects the mucous membranes of an exposed individual and then spreads to other parts of the body. Measles is known to infect only humans with no known animal reservoir [1, 2]. Measles has an incubation period of about 10 days (with a range of 7 to 18 days). It is characterized by prodromal fever, conjunctivitis, coryza, cough and presence of Koplik spots [3]. The mortality rate for measles infection in children is usually 0.2%, but may be up to 10% in malnourished children [1, 4]. In cases with complications, the mortality rate may rise to 20-30% [5].

Measles affects up to 20 million people a year worldwide, most of these infections are seen in the developing areas of Africa and Asia [1, 6]. Among the childhood vaccine-preventable diseases, measles causes the most deaths in children [7]. Globally, measles mortality fell 60% from an estimated 873,000 deaths in 1999 to 345,000 in 2005 [7]. Estimates for 2008 indicate deaths fell further to 164,000, with 77% of the remaining measles deaths in 2008 occurring within the Southeast Asian region [1, 8]. In 2014, measles infection resulted in about 73,000 deaths [9, 10]. Most measles morbidity and mortality were seen in under-five children [1]. As a result of widespread vaccination, the disease was eliminated from the Americas by 2016 [11]. The burden of morbidity and mortality of measles is thus reducing across the developed world. In 2013-14, there were almost 10,000 cases in 30 European countries. Most cases occurred in unvaccinated individuals [12].

In Africa, measles remains a leading cause of death and disability in most countries [13]. Early community-based studies have revealed measles case-fatality rates of 3%-34%, which is about 10-20 times those in industrialized countries [14]. In 2015, the World Health Organization estimated that of the 134,200 measles deaths recorded, majority were in sub-Saharan Africa [15]. High case-fatality rates in African and other developing countries are due to a young age at infection, poor shelter and overcrowding, underlying immune deficiency disorders due to malnutrition, vitamin A deficiency and lack of access to medical care. Before the introduction of measles vaccines, one-third of children in many African countries were infected in the first and second years of life and most children were infected before they reach 5 years of age [16]. In Africa, about 125 million preschool-aged children have vitamin A deficiency, placing them at high risk of death, severe infection, or blindness as a result of measles [17]. Mortality from measles increases during times of war or famine. In Ethiopia in 2000, measles was responsible for 22% of deaths in children less than five years of age and 17% of deaths in children aged 5-14 years [18].

Measles is an endemic disease in Nigeria, with recurrent outbreaks occurring at irregular intervals. Measles transmission in Nigeria occurs through all months of the year, but peaks in the dry season (February, March and April) [3]. Measles transmission also sometimes occurs immediately after the end of the rainy season and often reaches epidemic proportions in the dry season. Nigeria is one of only ten countries in the world with measles vaccine coverage of less than 50% [19, 20].

Nigeria is among the 45 countries that account for 94% of the deaths due to measles worldwide [1, 21]. There are few literature in Nigeria on the population-based prevalence of measles. However, some studies from tertiary hospitals showed the proportion of measles from pediatric admissions stands at between 1.3-5.1% [22, 23]. Furthermore, the reported CFRs for measles in Nigeria showed regional variations ranging from 1.9% to 12.4% [22, 23]. A study conducted in a secondary health center, in Southern Nigerian, observed that measles accounted for 3.1% of all pediatric admissions in the hospital; this figure is higher than the 2.3% reported in 1998 at a tertiary health center in the same city [24]. We conducted this study to epidemiologically describe measles infection in Nigeria and highlight the burden of the disease in the country, identify gaps and make recommendation for improvements on surveillance and control.

## Methods

### Study setting

Nigeria, with current estimated human population of about 180 million [25], is the most populous country in Africa and the seventh most populous country in the world. The country is located in west Africa [26]. Nigeria has 36 states and a Federal Capital Territory (FCT) with 774 Local Government Areas (LGAs), categorized into six geo-political zones (North-West, North-East, North-Central, South-West, South-East and South-South). Nigeria has more than 500 ethnic groups with Hausa, Yoruba and Igbo being the dominant ones. The rainy season in Nigeria starts between March and May and ends between September or November, depending on the regions. The dry season starts between October and December, and ends between April in some parts and may extend to May or June in other areas. Measles transmission in Nigeria occurs through all months of the year, but peaks in the dry season (February, March and April) [3].

### Study design

A retrospective secondary data analysis of measles specific Integrated Disease Surveillance and Response (IDSR) records.

### Study population

We reviewed all reported measles cases (both suspected and confirmed) in the IDSR for the period of 2012 to 2016. IDSR weekly epidemiological data for the years under review was obtained from Surveillance Unit, Nigerian Center for Disease Control. The document contains recorded measles cases from the 36 States (plus the FCT) of Nigeria.

### Measles surveillance in Nigeria

Measles surveillance in Nigeria is through the IDSR platform. The IDSR is a national disease reporting platform, covering priority diseases from all health facilities across the country. Information flows from the health facilities, through the ward focal persons to the local government areas (LGA) disease surveillance and notification officers (DSNOs), to the States DSNOs and then to the Federal Ministry of Health. Feedback goes through the opposite direction. The IDSR collects information on disease cases and deaths, facility location and laboratory outcomes.

### Measles case definitions

**Suspected case:** any person with fever and maculopapular (non-vesicular) generalized rash and cough, coryza or conjunctivitis (red eyes) or any person in whom a clinician suspects measles.

**Laboratory confirmed case:** a suspected case with laboratory confirmation (positive serum IgM antibody for measles).

**Epidemiologically-linked or epidemiologically confirmed case** is a suspected case, which has contact with a laboratory confirmed case.

**Clinically confirmed:** a case that meets the clinical case definition and for which no adequate blood specimen was taken [27].

**Discarded case:** a suspected case that does not meet the clinical or laboratory definitions [27].

### Laboratory investigations

Venous blood samples of suspected measles cases were usually confirmed at reference laboratories located in Kaduna and Lagos States, using serological test for serum immunoglobulin M (IgM) antibodies specific for measles. These samples are usually pooled and transported to these references laboratories.

### Data management

We sorted, extracted and cleaned relevant variables from the IDSR line list, these included number of cases, number of deaths, location and laboratory results. We did Univariate analysis, which included frequencies and proportions, using Microsoft excel 2016 and Health-Mapper.

### Ethical consideration

We obtained approval from the Public Health Department of the Federal Ministry of Health, before we collected the IDSR data set. Ethical clearance was also obtained from the ethical committee of the Federal Ministry of Health.

## Results

A total of 131,732 cases of measles were reported between January 2012 and September 2016. Highest number of cases 57,892 (43.95%) were reported in 2013, followed by 2015 with 24,421 (18.54%) reported cases. The least number of measles cases 11,061 (8.4%) were reported in 2012. Katsina State recorded the highest number of cases 18,056 (13.7%) over the 5-year period, followed by Yobe and Sokoto States with 14,683 (11.1%) and 13,330 (10.1%) respectively. Kwara State recorded the least number cases 375 (0.3%) over the same period. A total of 817 reported measles deaths were recorded within the same period, which gives a case fatality rate (CFR) of 0.62%. Highest CFR (1.43%) was recorded in 2012, followed by 2013 with (0.6%). The least CFR (0.44%) was recorded in 2016 (Table 1).

**Table 1:** yearly distribution of number of cases, number of deaths, attack and case fatality rates for measles cases in Nigeria from January 2012 to September 2016

Year	Cases	Percentage	Death	CFR
2012	11,061	8.40%	158	1.43%
2013	57,892	43.95%	348	0.60%
2014	15,989	12.14%	85	0.53%
2015	24,421	18.54%	127	0.52%
2016	22,369	16.98%	99	0.44%
**Total**	**131,732**	**100.00%**	**817**	**0.62%**

The North-west region recorded the highest measles attack rate (AR) 149.73 cases per 100,000 population within this period, while the South-south geopolitical region had the least AR with 15.81 cases per 100,000 population. The CFR per regions of Nigeria showed the North-central region having the highest with 4.38%, while the South-west region recorded the least CFR with 0.17% (Table 2).

**Table 2:** regional distribution of number of cases, number of deaths, attack rates and case

Regions	Projected Pop 2016	Cases	Deaths	AR/100,000 pop	CFR
North-west	45,667,564	68,380	429	149.73	0.63%
North-east	24,469,020	34,310	816	140.22	2.38%
North-central	26,819,540	10,051	440	37.48	4.38%
South-west	35,544,233	9,544	16	26.85	0.17%
South-east	20,622,822	5,498	202	26.66	3.67%
South-south	26,926,140	4,257	31	15.81	0.73%

Fatality rates for measles cases in Nigeria from January 2012 to September 2016

**Figure 1:**
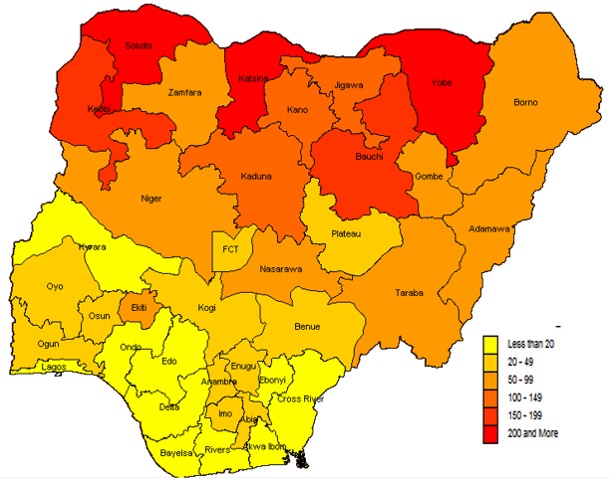
map of Nigeria showing measles attack rate per 100,000 population across the States, 2012 - 2016

**Figure 2:**
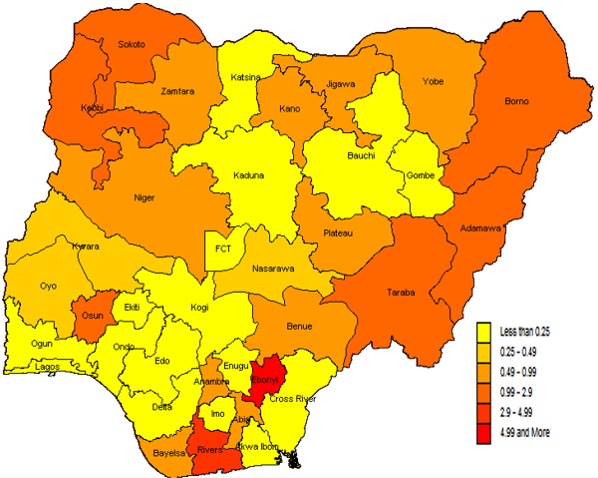
map of Nigeria showing measles case fatality rates (CFR) across the States, 2012 - 2016

Measles attack rates were higher in the northern States of Nigeria, with Katsina, Sokoto and Yobe, recording more than 200 cases per 100,000 population. While Kebbi and Bauchi recorded between 150 - 199 cases per 100,000 population. Most States in the southern regions recorded low attack rates for measles during the same period, with only Ekiti State recording between 50-99 cases per 100,000 population (Figure 1). Conversely, the measles CFR per States showed a different picture. Though more States from the northern region had higher CFR, two states from the southern region (Ebonyi and Rivers States) recorded the highest CFR (above 4.99%). Also, Katsina, Kaduna, Bauchi and Gombe States, all from the northern region, recorded CFR of less than 0.25% (Figure 2).

**Figure 3:**
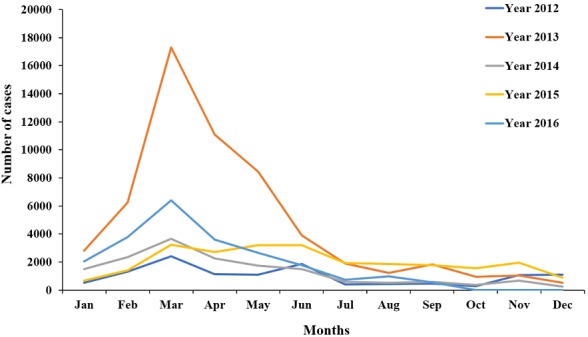
comparative yearly trend of measles cases in Nigeria from 2012 to 2016

**Figure 4:**
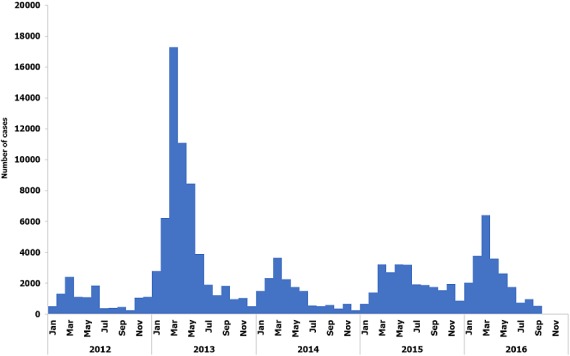
epidemic curve showing monthly occurrence of measles cases over a six-year period, from January 2012 to September 2016

The trend of measles cases followed the same pattern throughout the whole five years. Measles cases rose from February each year, reached a peak in March, then gradually reduced to the lowest level in June, which was maintained throughout the year. The year 2013 had the highest number of cases for each month, except for August, October, November and December, when it had comparatively lower number of cases than the other years (Figure 3). Measles cases were reported throughout the whole five-year period with no break in reporting, following similar patterns. Each year (from 2012 to 2016) showed a peak number of reported cases in March (Figure 4).

Of the 131,732 cases recorded within the study period, only 8,916 (6.7%) were confirmed by laboratory test, while 68,756 (52.2%) were confirmed by both clinical and epidemiological linkage. The highest proportion of laboratory confirmed cases (12.1%) was in 2012, followed by 2013 (6.7%), while the least was in 2015 with 5.7% of cases confirmed by laboratory tests (Table 3).

**Table 3:** yearly distribution of confirmed and laboratory confirmed measles cases in Nigeria from January 2012 to September 2016

Year	Total No of Cases	Confirmed (Epi-linked & clinically)		Laboratory Confirmed	
		Cases	Proportion	Cases	Proportion
2012	11,061	5,059	45.7%	1,338	12.1%
2013	57,892	40,321	69.6%	3,893	6.7%
2014	15,989	5,890	36.8%	974	6.1%
2015	24,421	8,271	33.9%	1,399	5.7%
2016	22,369	9,215	41.2%	1,312	5.9%
**Total**	**131,732**	**68,756**	**52.2%**	**8,916**	**6.7%**

## Discussion

We conducted this study to epidemiologically describe measles infection in Nigeria and highlight the burden of the disease in the country. Our study found a high burden of measles especially in the northern part of Nigeria. The high number of measles cases recorded over the five-year period in this study is worrisome when we take into account the availability of free and effective measles vaccines in the country, which is periodically given to children during routine and supplemental immunization activities [28]. This finding is also not in tune with measles control, because the ultimate global goal now is to eradicate measles. However, the earliest stage in measles eradication is measles control which involves reduction in mortality and morbidity. Following measles control is the elimination stage. Many African countries including Nigeria are at the measles control stage [1].

Measles attack rates in Nigeria from this study showed to be more in the northern regions, with several States from these regions severely affected than the southern States. These findings are in tune with findings from several studies that have opined measles infection to be concentrated in the northern region of Nigeria [3, 22, 24]. But, this study found measles CFR to be more in some southern States which contradicts findings from similar studies that found measles CFR to be consistently higher in the northern regions of Nigeria [22, 24]. This may be related to the relative higher number of SIAs in the northern region and improve measles case management due to an enhanced political commitment by most States government from the northern parts of Nigeria [29, 30].

The five-year CFR (0.6%) found in this study was lower than the CFR range of 3-5% been reported for developing countries globally [1, 31]. The reason for this may be related to the persistent yearly national measles immunization campaigns and improved measles surveillance in Nigeria. Also, the trend of CFR over the five-year period was decreasing, which was in tune with current trend of measles mortality reduction in Africa and across the globe [1]. This might have been due to improved surveillance, early case presentation and improved case management [32]. Our study revealed peak number of measles cases in first quarter of the year with no break in reporting for the five-year period in keeping with measles epidemiology. Similar patterns have been reported in the country and the WHO Africa region [1]. Only 6.7% of all reported measles cases were confirmed by laboratory diagnosis. This reflects that laboratory confirmation of measles is still very low in the country where all suspected measles cases are expected to be confirmed by laboratory testing.

### Limitations

Dataset was obtained without age and gender segregations, these have affected detailed description of the measles burden in Nigeria.

## Conclusion

The trend of measles in Nigeria over the 5-year period tends to be declining, but the burden still remains relatively high. Measles remains a major cause of childhood mortality and morbidity in Nigeria especially in the northern part of the country. Though measles case fatality has been on the decline over the years in review, laboratory confirmation of cases has been dismally low. Case-based surveillance provided an insight into understanding the epidemiology of measles infection in Nigeria.

**Recommendations:** 1) we recommended improvement on enhanced measles surveillance and routine immunization especially in the northern regions of Nigeria; 2) There is a need to work out alternate strategies for control of measles such as introducing a two-dose schedule to halt the endemic transmission (which has been adopted in some developing countries); 3) Improved measles case management across all regions; 4) Encourage blood sample collection and testing, strengthening and upgrading of States and regional laboratories to be able to perform confirmatory testing for measles, if the goal towards measles elimination is to be achieved.

### What is known about this topic

- Measles infection, despite being a vaccine preventable disease has continued to spread in most African countries, with devastating effects on mostly under 5-year children;
- The burden of measles in Nigeria has shown to be persistently higher in the northern region of the country.

### What this study adds

- Though mortality for measles in Nigeria was shown to be decreasing in trend over the study period, the burden has remained high over the study period;
- States in the southern region of Nigeria recorded highest measles mortality cases over the study period.

## Competing interests

The authors declare no competing interests.
